# Determination of UV Filters in Waste Sludge Using QuEChERS Method Followed by In-Port Derivatization Coupled with GC–MS/MS

**DOI:** 10.3390/mps5060092

**Published:** 2022-11-23

**Authors:** Cemile Yücel, Ilgi Karapinar, Serenay Ceren Tüzün, Hasan Ertaş, Fatma Nil Ertaş

**Affiliations:** 1 Faculty of Engineering, Environmental Engineering Department, Dokuz Eylül University, Tınaztepe Campus, Buca, İzmir 35390, Turkey; 2 Faculty of Science, Chemistry Department, Ege University, Bornova, İzmir 35040, Turkey

**Keywords:** UVFs, QuEChERS, in-port derivatization, waste sludge

## Abstract

UV filters (UVFs) are widely used in personal care and in industrial products for protection against photodegradation. In recent years, their potential toxicological and environmental effects have received growing attention. Due to their excessive use, their residue levels in the environment are gradually increasing and they tend to accumulate on biological wastewater treatment sludge. The utilization of sludge as fertilizer could be one of the main routes of UVF contamination in the environment. Therefore, the development of a reliable and sensitive method of analyzing their trace level residues in waste sludge samples is of great importance. The success of the method largely depends on the sample preparation technique in such complex matrices. This study presents a rapid, sensitive and green analysis method for eight UVFs in sludge samples, selected for their rather low no-observed-effect concentrations (NOEC). For this purpose, the QuEChERS methodology was coupled with in-port derivatization for subsequent detection of the targeted UVFs via GC–MS/MS. The analysis time was substantially shortened using this method, and reagent utilization was also reduced. The method was validated in the sludge samples, and high recovery (66–123%) and low RSD values (<25.6%) were obtained. In addition, major contributing uncertainty sources and expanded uncertainties were determined.

## 1. Introduction

UVFs are the general name for the chemical group that absorbs ultraviolet light, through which the adverse effects of UV light are eliminated. UVFs can be grouped as inorganic, such as TiO_2_ or ZnO [1], and organic, mostly used in personal care products, as well as in plastics, automobile paints and rubber industries to increase resistance towards UV light degradation [2]. These compounds can reach to water bodies through industrial and domestic effluents. Since their utilization in personal care products and industrial applications are widespread, the UVF residue levels in the environment are increasing extensively [3].

Organic UVFs are a wide range of compounds that differ in their structures and properties. These highly persistent compounds in the environment tend to accumulate in water [3,4,5], suspended particles, soil [6], sediment [7] and sludges [8,9,10]. Organic UVFs can also accumulate in biota and disrupt the endocrine systems of aquatic organisms by increasing their estrogen-induced cell proliferation [11]. It has been clearly stated that UVFs affect different hormonal targets as well as estrogenic activity in mammals and fish, and are therefore known as endocrine-disrupting chemicals (EDCs) [12,13]. Consequently, one of these compounds has recently been included in the Watch List by The European Water Framework Directive as a future priority pollutant [14]. Although the use of these compounds in cosmetics is legally restricted [15], there is no limit to their concentration in water and sewage sludge matrices. However, studies on the presence of UVFs in waste sludge have revealed that their concentration range from a few to thousands of µg/g in dry mass [16]. The use of this sludge as fertilizer is another concern due to the widespread distribution of UVF residues in agricultural soils and the possible contamination of crops.

Since their concentrations are very low, an appropriate preparation technique must be applied to the samples to isolate and preconcentrate these filters. Current trends in the determination of organic UVFs in environmental water samples based on microextraction techniques have been reviewed [3,17,18]. However, their determination in sludge samples faces difficulties due to the complexity of the matrices and their impact on the environment. To date, one of the techniques used for UVF extraction from sewage sludge comprises liquid–liquid extraction (LLE) followed by solid-phase extraction (SPE) [19,20,21,22] where the excessive use of solvent is the main drawback of the method. The other technique is pressurized liquid extraction (PLE), applied alone or coupled with SPE [23,24,25,26,27,28,29] or gel permeation chromatography (GPC) [30]. Despite the advantage of using a smaller solvent volume in the PLE technique, special equipment is required to conduct this method.

In the last few decades, method development studies have been devoted to modern sample preparation techniques based on shorter analytical periods and the minimization of organic solvent utilization for a wide range of pollutants. One of these modern techniques, called QuEChERS (which is an acronymic for quick, easy, cheap, effective, rugged and safe) has been well-established to improve laboratory efficiency and throughput. The main advantage of this method is its small quantities of solvent utilization. The method has already been tested for UVFs in human milk [31] and seafood samples [32], followed by liquid chromatography systems coupled with mass detectors (LC-MS) and sludge samples, and then, by detection using gas chromatography (GC–MS) [16,21,22,23]. LC-MS systems are preferred for the analysis of polar UVFs, and no derivatization step is required in accordance with the physicochemical properties of UVFs [33,34,35,36]. GC–MS has been used for rather non-polar and volatile UV filters. Although the GC method provides high separation efficiency, high selectivity and good sensitivity, it displays some disadvantages such as the derivatization step, in which more reagents, greater reaction time and more labor are required. Fortunately, the in-port derivatization technique is a practical and environmentally friendly solution to these issues wherein the reaction takes place in the injection block rather than in an off-line interaction. The analysis time can be substantially reduced using this method and the selectivity can be improved since the technique enables extra purification for the matrix effect.

In the present study, we aimed to detect eight UVFs in sludge samples. Benzophenone-3 (BP-3), 3-benzylidene camphor (3BC), 2-ethyl hexyl-4-(dimethyl amino) benzoate (EDP), 2-ethyl-hexyl-4-trimethoxy cinnamate (EHMC), ethylhexyl salicylate (EHS), homosalate (HMS), isoamyl p-methoxycinnamate (IAMC) and 4-methylbenzylidene camphor (4-MBC) were selected according to their no-observed-effect concentration (NOEC) values. Their chemical structures and NOEC limits are presented in Table 1. Considering the hydrophobicity (logK_ow_: 3.79–6.16) of the analytes, the QuEChERS method was adopted; however, due to the low volatility of these analytes along with their weak acidity (pK_a_: 7.56–8.13), the analytes were derivatized using an in-port silylation technique prior to subsequent quantification using the GC–MS/MS system. To the best of our knowledge, this is the first report in the literature about the combination of QuEChERS with in-port derivatization followed by GC–MS/MS for a wide variety of UVF analyses in waste sludge. This method extensively decreases solvent or chemical consumption and shortens the analysis time compared to off-line derivatization. The method validation parameters, major contributing uncertainty sources and expanded uncertainties were determined.

## 2. Materials and Method

### 2.1. Reagents and Standard Solutions

The chemical standards of 2-ethylhexyl salicylate (EHS), 2-hydroxy-4-methoxybenzophenone (benzophenone-3, BP-3) >98%, 3,3,5-trimethylciclohexylsalicylate (homosalate, HMS) >98%, 4-methylbenzylidene camphor (4-MBC) 99%, 2-ethyl-hexyl-4-trimethoxy cinnamate (EHMC) 99%, and 2-ethyl hexyl-4-(dimethylamino) benzoate (EDP) 99% were obtained from Dr. Ehrenstorfer (Augsburg, Germany). 3-benzylidene camphor (3BC) 99%, isoamyl p-methoxycinnamate (IAMC) and % were acquired from Sigma-Aldrich (St. Louis, MO, USA), Toronto Research Chemicals (Toronto, ON, Canada) and Chemservice (West Chester, PA, USA), respectively.

LC-MS-grade methanol and acetonitrile, GC–MS-grade ethyl acetate (EtAC) and acetone, sodium chloride, sodium sulfate and orthophosphoric acid were purchased from Merck (Darmstadt, Germany). Bis(trimethylsilyl) trifluoroacetamide with 1% trimethylchlorosilane (BSTFA + TMCS; 99:1, *v*/*v*) from Macherey-Nagel was used as derivatization reagent. This reagent was stable for only 3 h during analysis. 2-dodecanol, 2-dodecanone, and 1-undecanol from Sigma were used as extraction solvents. Anhydrous magnesium sulphate (MgSO_4_), primary and secondary amine exchange bonded silica sorbent (PSA) and octadecylsilan (C18) were obtained from Supelco (Bellefonte, PA, USA) for the extraction step.

The stock solutions of individual UVFs (1000 µg mL^−1^) and mixtures of UVFs (50 µg mL^−1^) were prepared in ethyl acetate to optimize the injection port derivatization conditions in methanol to validate the QuEChERS method. These solutions of 1000 µg mL^−1^ and 50 µg mL^−1^ were stable for about 5 months and five days at −20 °C, respectively. The working aqueous solutions were prepared daily from standards in methanol at different concentrations using ultrapure water and environmental water. The sludge samples were collected from a domestic wastewater treatment plant in Izmir, Turkey. Sludges were air-dried and stored in the dark at −20 °C until analysis.

### 2.2. Sample Extraction

**Figure 1:**
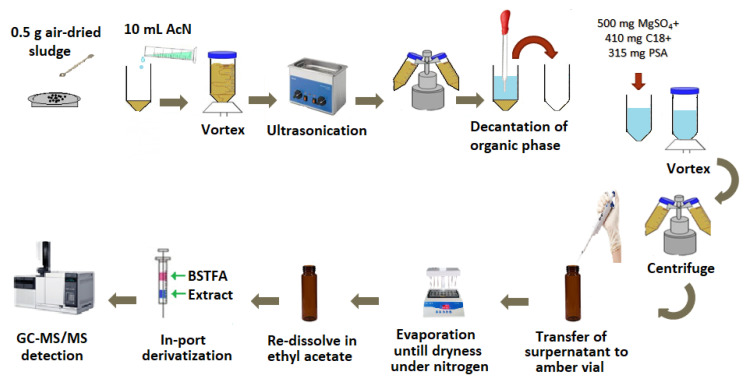
Schematic illustration of the whole procedure.

The procedure for extracting UVFs from sludge is a modified version of the method reported previously [16]. As shown in Figure 1, a 0.5 g sewage sludge sample was dried at room temperature and a spiked sample was transferred into a conical-bottom 15 mL polypropylene tube containing 10 mL of ACN, vortexed for 2.5 min, and then, left in an ultrasonic bath (J.P. Selecta, Barcelona, Spain) for 15 min. Then, the organic phase was separated via centrifugation at 3500 rpm for 15 min and transferred to a conical-bottom 15 mL polypropylene tube containing 500 mg MgSO_4_, 410 mg C18 and 315 mg PSA. The extract was then vortexed for 2.5 min and centrifuged for 15 min. After the supernatant was transferred to a 10 mL tube, it was evaporated to dryness under N_2_ gas. Then, the extract was dissolved in 1000 µL EtAC, instead of hexane as performed by Ramos et al. [16], for the in-port derivatization step as previously applied to a surface water sample by our research group [37]. The extract dissolved in EtAC was filtered through a 13 mm, 0.22 µm PTFE filter, and then, it was transferred to a 1.5 mL amber vial. Finally, 2 µL of BSTFA and 2 µL of extract were derivatized in the injection port using the sandwich technique. In this technique, two aliquots of 2 µL BSTFA and 2 µL extract in EtAc, separated with an air gap, are drawn into the microsyringe of a PAL autosampler, and then, injected to the GC–MS/MS system.

### 2.3. GC–MS/MS Analysis

GC–MS/MS analysis was performed using an Agilent 7890 B gas chromatograph coupled with a triple quadrupole mass spectrometer (MS 7000C) and a PAL autosampler (GC Sampler 80). For derivatization, the injection port temperature was held at 70 °C for 3 min, then, increased to 300 °C at a rate of 400 °C min^−1^ in splitless mode with a purge-off time of 4.5 min. The oven temperature started at 70 °C for 4 min, increased to 180 °C at 25 °C min^−1^, then, increased to 230 °C at a rate of 5 °C min^−1^ and to 300 °C at a rate of 25 °C min^−1^; it was held for 10 min at this temperature. The injector was operated using programmed temperature evaporation (PTV).

Separation was performed on 5% phenyl-arylene/95%-dimethylpolysiloxane HP-5MS (30 m × 0.25 mm i.d. 0.25 µm film thickness) supplied by Phenomenex (Torrance, CA, USA). Helium (99.999% purity) was used as the carrier gas at a constant flow of 1 mL min^−1^. In MS/MS analysis, the temperatures of the ion source and the transfer line were 280 and 300 °C, respectively. The multiple reaction monitoring (MRM) technique was applied, and electron ionization (EI) mode was used. The retention times (R_t_) obtained, the optimized MRM transitions and the collision energies (CE) for each UVF are given in Table 2. The bold parent and product ions in the table show the quantification transitions.

### 2.4. Validation Studies

The matrix match method was used for the calibration curves. In this method, the curves were constructed by subtracting the peak area values of the real sludge sample from the spiked extract and plotting against the concentration of the UV filter added into the real sludge sample.

The linear range, intra-day and inter-day repeatability, Limit of Detection (LOD), Limit of Quantitation (LOQ) and recovery parameters were determined, and measurement uncertainties of the method applied for UVFs were calculated. For the linearity of UVFs, sludge samples were spiked with 40, 80, 200, 600 and 1200 ng g^−1^ UVF standards. The extracts were evaporated to dryness under nitrogen gas, and they were diluted to 1000 µL using ethyl acetate. The LOQ and LOD were calculated according to S/N = 10 and S/N = 3, respectively. Intra-day and inter-day repeatability studies were performed at low (80 ng g^−1^), medium (300 ng g^−1^), and high (600 ng g^−1^) concentrations, with 3 replicates.

The selected test material was analyzed repeatedly under different conditions such as on different days, using different analysts and different equipment, etc. The total variation in the whole cluster can be represented as the combination of variances (s^2^) between (S*_between_*) and within groups (S_r_). The repeatability (intra-day) and intermediate precision (inter-day) values were calculated via ANOVA [38]. The standard deviation of S_r_ was calculated by taking the square root of the within-group mean square term, as shown in Equation (1), and the contribution of the grouping factor to the total variation was obtained from Equation (2). In Equations (1) and (2), MS_w_ is the within-group mean square term and *MS_b_* is the between-group mean square term.

Then, intermediate precision (S_I_) was calculated by combining the within-group and between-group variance components, as shown in Equation (3).

(1) $$S_{r}=\sqrt{MS_{w}}$$

(2) $$s_{between}=\sqrt{\frac{MS_{b}-MS_{w}}{n}}$$

(3) $$S_{I}=\sqrt{S_{r}{}^{2}+S_{between}{}^{2}}$$

The intra-day (*n* = 3) and inter-day (*n* = 2) relative standard deviations (RSD%) of the QuEChERS followed by GC–MS/MS were obtained using spiked solutions of the analytes at different concentration levels. According to the 2015/1787 directive, if the RSD% value of the applied method is less than 25%, the precision of this method is acceptable for organic compounds.

Recovery studies to determine the accuracy of the method were carried out by adding the UVF standards to the sewage sludge samples at concentrations of 80, 300 and 600 ng g^−1^. The recovery percentages were calculated from Equation (4) where, Cpre−Ext and Cpost−Ext are the concentrations of sludges in which analytes were added before and after extraction, respectively. Csample is the concentration of UVFs in sludges without the addition of the analyte.

(4) $$Recovery\;\%=\frac{Cpre-Ext-Csample}{Cpost-Ext-Csample}\times 100$$

The measurement uncertainty is a parameter that was included with the measured result and characterizes the distribution of values that can correspond to the measurand. Knowing the uncertainty means increased confidence in the accuracy of the measurement result. This value is very important in comparing the measurement results of two different methods and deciding whether the results are within the defined limits. Measurement uncertainty consists of many components. Some of these components are derived from the statistical distribution of the results of repeated measurement series to obtain the standard deviations. Combined standard uncertainty (*u*(*c*)) is the standard uncertainty that considers contributions from all important uncertainty sources by combining the relevant uncertainty components, and it was calculated as shown in Equation (5). The expanded uncertainty provides the range of an analyte concentration believed to be spread at a higher confidence level. The expanded uncertainty (*U*) is calculated by multiplying the combined standard uncertainty by “*k*”, which is equal to 2 at 95% confidence level [39]. In this study, the uncertainty sources were defined first; then, the uncertainty of each parameter was determined, and finally, the combined *U*c(y) and expanded uncertainties (*U*) were calculated.

(5) $$u\left(c\right)=\sqrt{u_{calibration}^{2}+u_{SI}^{2}+u_{Recovery}^{2}}$$

## 3. Results and Discussion

### 3.1. Optimization Studies

**Figure 2:**
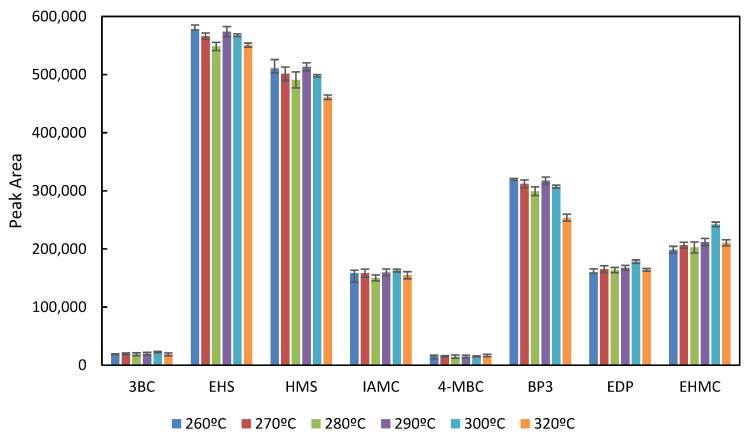
The effect of injection temperature on peak areas of UVFs.

For the extraction of targeted UVFs from sludge samples, the QuEChERS methodology was adopted for the in-port derivatization and the extract obtained was dissolved in 1000 µL ethyl acetate (EtAc) instead of hexane. In a previous study carried out in this lab, in-port derivatization conditions were optimized for a wide range of UVFs extracted from surface water via vortex-assisted dispersive liquid–liquid microextraction based on the solidified floating organic droplet (VA-DLLME-SFOD) technique. It was determined that the injection temperature was a statistically significant factor and the optimal temperature was determined to be 260 °C [37]. The effect of injection temperature for the studied UVFs in sludge samples was further studied to see any deviation from the optimal conditions determined. The temperature varied between 260–320 °C, and the peak areas are given in Figure 2.

**Figure 3:**
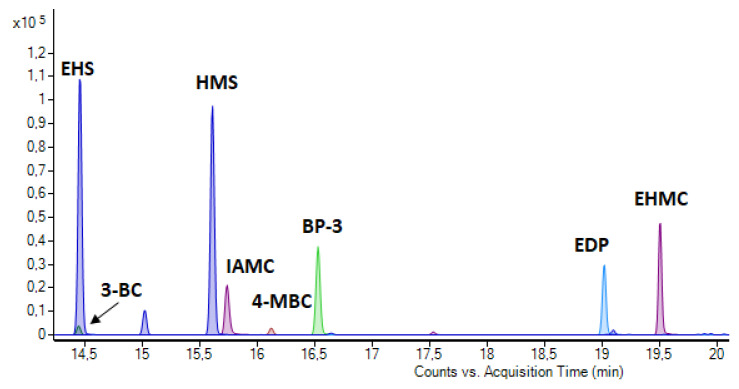
Chromatograms of the UV filters in sewage sludge spiked with UVFs at 300 ng g^−1^.

As can be deduced from the figure, even though the mean peak areas of the UVFs are not substantially different from each other for the studied injection temperatures, a slight decrease was observed for 300 °C. However, this injection temperature was chosen for further studies since the carry-over effect occurred in samples up to 290 °C. The sharp peaks in UVFs with injection port derivatization, after QuEChERS, of the spiked sludge sample were obtained, as shown in Figure 3.

### 3.2. Validation Studies

The linearity and linear range of a method should unequivocally be determined for any analytical method. It is a fact that the lowest concentration of the calibration curve should be very close to the LOQ value for the accurate analysis of analytes with known precision at an LOQ-level concentration. Table 3 depicts the linear equations for the working range of 40–1200 ng g^−1^ with correlation coefficients close to unity (R^2^ > 0.9970).

LOQ is defined as the lowest concentration that can be measured with acceptable precision (20% RSD) and accuracy [40,41,42,43,44,45]. Three different methods are used to determine the LOQ value. The most common approach for LOQ calculation in chromatographic analysis is the signal-to-noise ratio (S/N) [45]. This ratio can be defined as the difference between the height of the analyte peak (signal) and the highest and lowest points of the baseline (noise) in each area around the signal. For LOQ, S/N usually needs to be at least equal to 10. In the second method, a specific calibration curve should be studied using samples containing an analyte in the range of the LOQ. The residual standard deviation of a regression line or the standard deviation of the y-intercepts of regression lines may be used as the standard deviation [39]. The third approach is the concentration corresponding to a response 10 times greater than the SD of the analysis at the minimum concentration [37,41,45].

The LOD and LOQ values were calculated based on the S/N = 3 approach and S/N = 10, respectively. The LOQ values calculated using this method were found to be in agreement with previous studies on the analysis of UVFs in sludge [17,24,26]. In-port derivatization provides lower detection limits by converting polar analytes to more volatile compounds, as reported earlier [46,47,48]. For most of the compounds, the LOQ is close to or less than the lowest concentration level of the calibration curve. The RSD% values obtained in this study are below 25% as seen in Table 4.

The recoveries were in the range of 66%–23% as depicted in Table 5. Although, the recoveries are almost the same as the results obtained in other studies [17,24,26,31], our method provides certain advantages such as the lack of special apparatus and comparatively shorter analysis times.

### 3.3. Measurement Uncertainty

Here, the main uncertainty components such as *u_calibration_*, *u_SI_* and *u_Recovery_* were calculated for the analysis of two different UVF concentrations, as given in Table 6. The uncertainty of the QuEChERS-GC–MS/MS method for UVFs is between 13.2–47.4% at a concentration of 300 ng g^−1^ and between 6.9–43.6% at a concentration of 600 ng g^−1^, which are quite satisfactory in such a complex matrix. Uncertainties regarding the recovery and calibration curve were the largest source contributing to measurement uncertainty.

### 3.4. Comparison with Other Methods

The recovery values obtained for all analytes are similar to those of studies with laborious and expensive techniques. In some studies, recovery values at a single concentration were determined [21,22,27,36], while in others, recovery values at different concentrations were investigated [16,19,23,33]. Our results indicated that for 4-MBC, only at a high concentration (600 ng g^−1^) was the recovery value lower than that of the reported ones. Fortunately, good recovery values were obtained at lower concentrations. In addition, to the best of our knowledge, method validation for the analysis of 3-BC, EHS, HMS and IAMC in sludge was attained successfully. Analytes (EHS, HMS, BP-3) were derivatized using the in-port derivatization technique, which is fast, reliable and eco-friendly without being affected by the complexity of the matrix.

The LOD values of the selected UVFs in the spiked sludge sample ranged from 8 ng g^−1^ to 12.1 ng g^−1^. These limits are quite successful when compared to other studies (Table 7). The LOD values obtained for UVFs were lower than previous studies for 4-MBC, EHMC [23,27], BP-3 [23,36] and EDP [16]. On the other hand, the LOD values for 4-MBC [21,22,33], EHMC [16,21,22], BP-3 [19,27,33] and EDP [23] were higher.

The developed method was applied to sludge samples collected from a domestic wastewater treatment plant. The concentrations of UV filters were found to be 66.9, 161.9, 54.5, <LOQ and 79.9 ng g^−1^ for EHS, HMS, BP-3, EDP and EHMC, respectively. These results indicate that even in domestic wastewater sludge, high concentrations of UVFs could be found, and the sludge used for soil conditioning or fertilization purposes could raise environmental concerns.

## 4. Conclusions

In this study, eight different UVFs were extracted from sludge samples using a well-established QuEChERS methodology, and then, in-port derivatization was applied for more polar and less volatile analytes. The method combining QuEChERS and in-port derivatization, which ensures extra sensitivity, was shown to be accurate, reproducible and sensitive. This method also provided substantially reduced analysis time and solvent or chemical consumption compared to off-line derivatization. Method validation resulted in high recovery of UVFs (66–123%), meaning good accuracy; moreover, low inter-day RSD% (10.1–25.6) indicates high precision, and low values of LOD (<2.1 ng g^−1^) and LOQ (<40 ng g^−1^) show the sensitivity of the analysis for a matrix as complex as waste sludge. This study can be extended to the analysis of UVF degradation metabolites in sludge samples using QuEChERS with in-port derivatization followed by GC–MS/MS.

## Figures and Tables

**Table 1:** Chemical Structures, IUPAC names and NOEC concentration of selected UVFs.

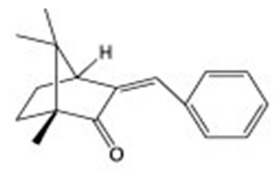 3-BC: 3-benzyline camphor (3-benzylidene-1,7,7-trimethylbicyclo [2.2.1] heptan-2-one) NOEC: 0.022 mg L^−1^	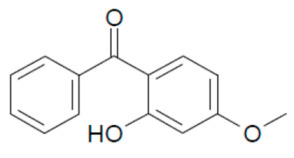 BP-3: benzophenone-3 (2-hydroxy-4-methoxy phenyl)-phenyl methanone NOEC > 0.01 mg L^−1^
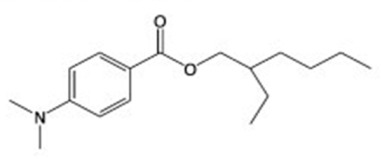 EDP: 2-ethyl hexyl-4-(dimethylamino) benzoate NOEC: 0.012 mg L^−1^	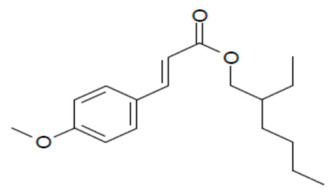 EHMC: 2-ethyl-hexyl-4-trimethoxy cinnamate NOEC: 0.003 mg L^−1^
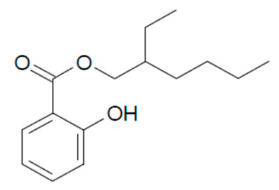 EHS: 2-ethylhexyl 2-hydroxybenzoate (ethylhexylsalicylate) NOEC: 0.008 mg L^−1^	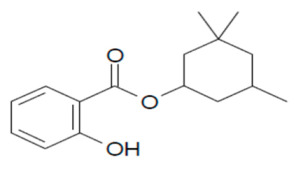 HMS: 3,3,5-trimethylcyclohexyl 2-hydroxybenzoate (Homosalate) NOEC: 0.005 mg L^−1^
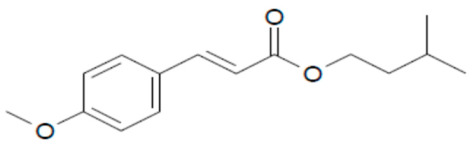 IAMC: isoamyl p-methoxy cinnamate (3-methylbutyl (2E)-3-(4-methoxyphenyl) acrylate) NOEC: 0.013 mg L^−1^	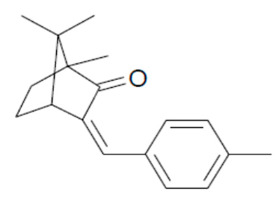 4-MBC: 4-methylbenzylidene camphor NOEC: 0.008 mg L^−1^

**Table 2:** Experimental GC–MS/MS parameters of UVFs.

UVFs	R_t_ (min)	MW (g mol^−1^)	Parent Ions (*m*/*z*)	Product Ions (*m*/*z*)	CE (eV)
3-BC	14.443	240.35	**240.0**	**149.2** 225.1 92.10	5 9 12
EHS	14.452	250.34	**195.0**	**177.0** 159.0 75.00	15 25 30
HMS	15.610	262.36	**195.0**	**177.0** 159.0 75.00	15 25 27
IAMC	15.737	248.32	**178.1** 161.0	**161.1** 133.0	15 10
4-MBC	16.120	254.37	**254.0**	**239.0** 105.0	15 25
BP-3	16.527	228.25	**285.0**	**242.0**	25
EDP	19.014	277.41	**277.0** 148.0 165.0	**164.9** 104.2 148.6	10 30 32
EHMC	19.501	290.41	**161.0** 178.0 290.0	**133.1** 133.1 178.1	8 22 6

**Table 3:** Analytical merits of the GC–MS/MS method coupled with QuEChERS for UVF determination in sludge samples.

Analyte	a *	b *	R^2^	LOD (ng g^−1^)	LOQ (ng g^−1^)
3-BC	30.154	−591.4	0.9991	10.1	39.6
EHS	888.02	−23,900	0.9987	10.0	33.3
HMS	515.64	−11,424	0.9984	9.90	33.0
IAMC	170.2	−285.3	0.9996	9.50	31.8
4-MBC	24.748	−159.2	0.9997	8.00	26.7
BP−3	276.81	−10,099	0.9980	4.30	14.1
EDP	214.00	−1290	0.9996	12.1	40.2
EHMC	204.46	−3367	0.9970	8.00	26.8

* Y = ax + b.

**Table 4:** Intra-day (*n* = 3) and inter-day (*n* = 2) relative standard deviations (RSD%) calculated for the UVFs.

UVFs	Intra-Day RSD% (*n* = 3)			Inter-Day RSD% (*n* = 2)		
	80 ng g^−1^	300 ng g^−1^	600 ng g^−1^	80 ng g^−1^	300 ng g^−1^	600 ng g^−1^
3-BC	4.3	8.7	3.2	10.8	9.1	7.1
EHS	9.9	9.7	17.2	10.9	10.9	19.3
HMS	13.1	12.1	21.9	19.8	13.7	25.6
IAMC	5.2	8.2	3.4	15.1	17.9	19.8
4-MBC	4.0	8.1	3.2	10.1	10.7	7.2
BP-3	16.2	11.6	13.6	17.7	13.7	15.5
EDP	4.9	11.2	2.3	15.0	14.4	15.4
EHMC	9.2	12.9	3.4	22.6	15.9	18.8

**Table 5:** Recoveries of UVFs from sludge samples spiked with different concentrations.

UVFs	% Recovery ± RSD (*n* = 3)		
	80 ng g^−1^	300 ng g^−1^	600 ng g^−1^
**3-BC**	115 ± 4.49	93 ± 10.1	98 ± 4.03
**EHS**	113 ± 6.83	98 ± 10.2	96 ± 7.34
**HMS**	87 ± 11.1	109 ± 12.5	104 ± 25.8
**IAMC**	121 ± 2.70	118 ± 8.80	113 ± 4.70
**4-MBC**	97 ± 2.35	75 ± 9.12	66 ± 3.05
**BP-3**	88 ± 18.1	87 ± 14.9	66 ± 14.7
**EDP**	106 ± 2.25	103 ± 12.1	103 ± 3.21
**EHMC**	123 ± 7.49	108 ± 14.4	107 ± 4.43

**Table 6:** Expanded uncertainties of QuEChERS followed by the in-port derivatization method for studied UVFs.

UV Filter	Description	Value (ng g^−1^)*x*		Standard Uncertainty *u*(*x*)		Relative Standard Uncertainty *u*(*x*)	
		300	600	300	600	300	600
EHS	Repeatability	1	1	0.0559	0.0860	0.0559	0.0860
	Bias (recovery)	0.8700	0.9948	0.0336	0.0582	0.0385	0.0585
	Calibration	300	600	14.855	15.075	0.0495	0.0251
	*u*(*c*)					0.0841	0.1070
	Expanded *U*(*x*)					0.1681	0.2139
HMS	Repeatability	1	1	0.0696	0.1267	0.0696	0.12673
	Bias (recovery)	0.8367	0.9183	0.1719	0.0939	0.2054	0.1022
	Calibration	300	600	16.826	17.0757	0.0561	0.0284
	*u*(*c*)					0.2241	0.1653
	Expanded *U*(*x*)					0.4481	0.3306
3-BC	Repeatability	1	1	0.0502	0.0182	0.0502	0.0182
	Bias (recovery)	0.9064	0.9294	0.0361	0.0268	0.0398	0.0288
	Calibration	300	600	12.634	12.822	0.0421	0.0214
	*u*(*c*)					0.0767	0.0403
	Expanded *U*(*x*)					0.1534	0.0805
IAMC	Repeatability	1	1	0.0473	0.0198	0.0473	0.0198
	Bias (recovery)	0.9653	0.9527	0.0677	0.0756	0.0702	0.0793
	Calibration	300	600	8.2934	8.2934	0.0276	0.0138
	*u*(*c*)					0.0890	0.0829
	Expanded *U*(*x*)					0.1780	0.1658
4-MBC	Repeatability	1.0000	1	0.0465	0.0185	0.0465	0.0185
	Bias (recovery)	0.6423	0.6257	0.0262	0.0167	0.0408	0.0266
	Calibration	300	600	6.6834	6.7825	0.0223	0.0113
	*u*(*c*)					0.0657	0.0343
	Expanded *U*(*x*)					0.1315	0.0687
BP-3	Repeatability	1	1	0.0670	0.0784	0.0670	0.0784
	Bias (recovery)	0.8426	0.6435	0.0507	0.0342	0.0602	0.0531
	Calibration	300	600	18.7049	18.9821	0.0623	0.0316
	*u*(*c*)					0.1096	0.0998
	Expanded *U*(*x*)					0.2191	0.1997
EDP	Repeatability	1	1	0.0645	0.0135	0.0645	0.0135
	Bias (recovery)	0.8690	0.9033	0.0477	0.0505	0.0548	0.0559
	Calibration	300	600	14.1641	14.3741	0.0472	0.0240
	*u*(*c*)					0.0970	0.0623
	Expanded *U*(*x*)					0.1939	0.1246
EHMC	Repeatability	1	1	0.0747	0.0193	0.0747	0.0193
	Bias (recovery)	0.7552	56,847	0.1589	1.2119	0.2104	0.2132
	Calibration	300	600	23.9792	24.3341	0.0799	0.0406
	*u*(*c*)					0.2371	0.2179
	Expanded *U*(*x*)					0.4743	0.4357

**Table 7:** Comparison of QuEChERS method with previous microextraction studies in sludge samples.

UV Filters	Extraction Method	Instrumental Method	LOD (ng g^−1^)	Recovery (%)	RSD%	References
4-MBC OC EHMC ODP BP-3 BP-1 4HB 4DHB	PLE	UPLC-MS/MS	12 18 19 0.2 1.0 60 5.0 5.0	102 70 90 85 70 30 95 96	16–10 4–9 5–10 7 5–9 9–14 4–11 3–6	[27]
EHS HMS IAMC BP-3 4-MBC EDP EHMC OC	PLE + SPE	GC–MS	17 * 34 * 34 * 61 * 26 * 22 * 24 * 33 *	95–101 78–96 80–107 89–106 79–86 88–93 73–90 84–85	7 5–6 4–6 6–11 4–5 6–7 5 5–12	[23]
BP-1 BP-2 BP-3, BP-4 PBSA	PLE	LC-MS/MS	2.5 * 2.5 * 25 * 5 * 5 *	74 ± 9 99 ± 11 104 ± 14 114 ± 28 118 ± 19	9 11 14 28 19	[36]
BP-1, BP-2 BP-3 BP-8 1H-BT 5Me-1H-BT TBHPBT 4-OH-HB	LLE + SPE	LC-MS/MS	0.41 0.67 0.67 0.41 0.67 0.67 0.1 0.41	38.3–116	3.14–13.8	[19]
4-MBC EHMC OC	LLE-SPE	GC-MS	4 3 6	94.6 101.2 87.5	13.1 10.5 7.5	[21]
4-MBC EHMC OC	LLE-SPE	GC–MS	4 3 6	95 101 87	2 13 7	[22]
BP BP-3 4-MBC	QuEChERS	UPLC-MS/MS	0.3 0.3 0.6	63–82 60–86 86–95	0.1–1.0 0.2–0.5 0.1–6.0	[33]
BP 4-MBC EDP EHMC OC	QuEChERS	GC-MS/MS	26 59 31 5 6	92–101 85–88 82–86 113–125 81–94	2–6 2–7 1–2 1–5 3–5	[16]
3-BC EHS HMS IAMC 4-MBC BP3 EDP EHMC	QuEChERS	GC–MS/MS	10.1 10.0 9.90 9.5 8.00 4.30 12.1 8.00	93–115 96–113 87–109 113–121 66–97 66–88 103–106 107–123	3.2–10.8 9.7–19.3 12.1–25.6 3.4–19.8 3.2–10.7 13.6–16.2 2.3–15.4 3.4–22.6	This study

1H-BT: 1H-benzotriazole, 5Me-1H-BT: 5-methyl-1H-benzotriazole, TBHPBT: 2-(5-t-butyl-2-hydroxyphenyl) benzotriazole, 4-OH-HB: 4-hydroxy benzophenone. * LOQ values.

## Data Availability

Not applicable.
